# Nanoscale imaging of major and minor ampullate silk from the orb-web spider Nephila Madagascariensis

**DOI:** 10.1038/s41598-023-33839-z

**Published:** 2023-04-24

**Authors:** Irina Iachina, Jacek Fiutowski, Horst-Günter Rubahn, Fritz Vollrath, Jonathan R. Brewer

**Affiliations:** 1 Department of Biochemistry and Molecular Biology, University of Southern Denmark, Odense, Denmark; 2 Mads Clausen Institute, SDU NanoSYD, University of Southern Denmark, Sønderborg, Denmark; 3 Department of Biology, University of Oxford, South Parks Rd., Oxford, UK

**Keywords:** Biophysics, Biopolymers in vivo

## Abstract

Spider silk fibres have unique mechanical properties due to their hierarchical structure and the nanoscale organization of their proteins. Novel imaging techniques reveal new insights into the macro- and nanoscopic structure of Major (MAS) and Minor (MiS) Ampullate silk fibres from pristine samples of the orb-web spider *Nephila Madagascariensis*. Untreated threads were imaged using Coherent Anti-Stokes Raman Scattering and Confocal Microscopy, which revealed an outer lipid layer surrounding an autofluorescent protein core, that is divided into two layers in both fibre types. Helium ion imaging shows the inner fibrils without chemical or mechanical modifications. The fibrils are arranged parallel to the long axis of the fibres with typical spacing between fibrils of 230 nm ± 22 nm in the MAS fibres and 99 nm ± 24 nm in the MiS fibres. Confocal Reflection Fluorescence Depletion (CRFD) microscopy imaged these nano-fibrils through the whole fibre and showed diameters of 145 nm ± 18 nm and 116 nm ± 12 nm for MAS and MiS, respectively. The combined data from HIM and CRFD suggests that the silk fibres consist of multiple nanoscale parallel protein fibrils with crystalline cores oriented along the fibre axes, surrounded by areas with less scattering and more amorphous protein structures.

## Introduction

During the construction of its web, a typical orb-weaver spider uses two types of silk for the scaffolds of the structure that support the sticky capture spiral that holds any prey. The Major Ampullate silk (MAS) is used primarily for the radii spokes of the web, whereas the Minor Ampullate silk (MiS) is used principally for a temporary scaffold during web construction but also accompanies MAS silks in radii and safety lines, always dragged behind by the spider^1^. For several reasons MAS is the silk type most studied although MiS fibres are gaining scientific attention as they share gland morphology and some protein motifs with MAS fibres but display rather different mechanical properties^2–4^. Our comparisons of the two types of silk provide novel insights into functional relationships by demonstrating how small structural changes at the molecular level give rise to large differences in morphometric properties, which in turn can potentially be linked to fundamental structure–function relationships.

Here we focus on the structure of the innermost layer referred to as 'the protein core', which comprises a layered structure containing several different proteins^5,6^. The MAS fibres of the orb-weaver *Nephila clavipes* display an outermost lipid layer of 10–20 nm thickness surrounding the protein core^6,7^. The structure of the protein core is more difficult to image as it is (apparently) rather more complex. Attempts of visualization include Atomic Force Microscopy^8–12^, Light Microscopy^13^, Confocal Laser Scanning Microscopy^14^, Scanning Electron Microscopy and^11,14,15^, and Transmission Electron Microscopy^15,16^. All these techniques have their own drawbacks, which limit their data concerning full disclosure of the structural nature of spider silk while overall providing important insights. For example, TEM requires thin sectioning and thus the sample to be glutaraldehyde fixed or deep frozen. This in turn causes artefacts such as cavities and fractures within the silk fibres^15^. For AFM imaging the outer layers of the silk must be removed either mechanically or chemically^11^, as AFM only allows for surface imaging. Cross sectioning and subsequent AFM imaging reveal protein fibrils with a diameter of around 100–150 nm in MAS fibres from *Nephila clavipes.* However, for those measurements’ samples were also cryogenically frozen before imaging, fostering a misinterpretation of cavities as 'fibrils'^10^. Fibral widths of down to 50 nm have been reported for *Loxosceles laeta silk*^17^. In light microscopy studies of silk structure the samples were submerged in urea and studied under super contraction conditions, which supports unwanted delamination processes^18–20^. While all the microscopy observations are useful to dissect the structures of the silk, they also include artifacts, which taken on their own can lead to misinterpretations.

In this article, we focus on pristine, i.e., untreated spider silk fibres and report results obtained with alternative i.e. less invasive techniques such as Coherent Anti-Stokes Raman Scattering (CARS) microscopy^21,22^, Confocal Microscopy, Ultra-resolution Confocal Reflection Fluorescence Depletion Microscopy (CRFD)^23–27^ and Scanning Helium Ion Microscopy (HIM) as well as Helium Ion Sputtering^28–30^. These methods allow us to image the samples at near-physiological conditions, with minimal sample preparation and unprecedented resolution.

CARS microscopy enables us to image specific chemical bonds without labeling^21,22^, whereas confocal microscopy provides complementary information via labelling and subsequent imaging of specific molecules. However, both of these techniques are limited to a resolution of around 200 nm^31^. As the fibrils are expected to be around 150 nm in diameter^10,11^, we have applied (CRFD) microscopy^23–27^ and scanning Helium Ion microscopy (HIM)^28–30^. With the latter technique it is possible to achieve lateral resolutions on the order of 1 nm in spider silk. Using the sputtering option, removal of the outermost layers is possible^30,32,33^. CRFD microscopy is a novel combination of two optical techniques, allowing us to image the fibrils throughout intact fibres with a resolution of 100–120 nm^23–27^. Laurdan Generalized Polarization (GP) measurements^34,35^ in addition provide information on the phase of the lipids in the outermost layers of the silk.

In the following we present the data from investigations of pristine MAS and MiS fibres from the orb-weaving spider *Nephila madagascariensis*. We aim to obtain novel insights into the structure of spider silk at different size scales leading to a refined model for these fibres.

## Results

Visualization of the layered structure of spider silk was done using CARS microscopy. To demonstrate that spider silk fibres consist of protein layers surrounded by a lipid layer, CH_2_-stretches in intact MAS and MiS fibres were imaged with CARS microscopy.

**Figure 1:**
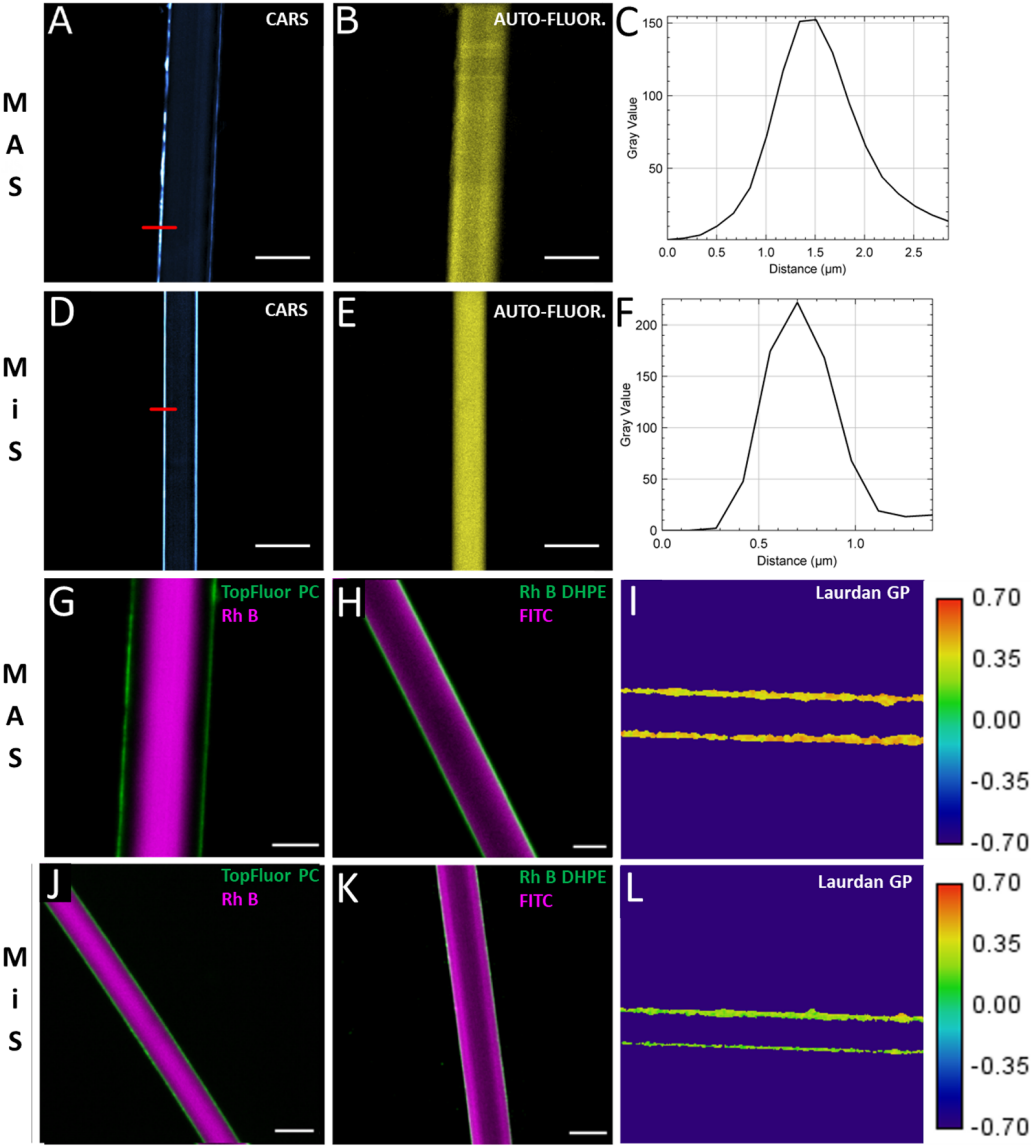
Images of optical sections taken at the middle of MAS and Mis fibres from the orb-weaver spider Nephila madagascariensis. (**A**) CARS image based on the CH_2_-stretch (2850 cm^−l^) vibration in a MAS fibre. (**B**) Image of autofluorescence. (**C**) Line profile of the lipid containing layer, see the red line in (**A**). (**D**) CARS image of CH_2_-stretch (2850 cm^−l^) in a MiS fibre. (**E**) Corresponding image of autofluorescence. (**F**) Line profile of the lipid containing layer measured along the red line in (**D**). Panels (**G**), (**H**), (**J**) and (**K**) are laser scanning confocal images of an optical section of MAS and MiS spider silk fibres labeled with TopFluor PC (green) and Rhodamine B (magenta) (**G**,**J**) and FITC (magenta) and Rhodamine B DHPE (green) (**H**,**K**). (**I**,**L**) Representative image of Laurdan GP of MAS (**I**) and MiS (**L**) silk fibre labeled with Laurdan, measured using two-photon excitation. Blue regions correspond to a low GP value (fluid phase) and green regions to a higher GP value (liquid-ordered phase) as seen on the color scale to the right. Scalebars are 10 µm.

Examining images of the CH_2_-stretch vibrations (Fig. 1A,C) in MAS and (Fig. 1D,F) MiS fibres reveal a very strong CARS signal at the edges of the samples.

Lipids typically exhibit a strong CARS signal due to a high density of emitters thus confirming an outermost layer containing lipids. The thickness of this layer has previously been found to be 10–20 nm using TEM^6^. Using the present CARS setup, the thickness of this lipid rich layer was measured at the equator of the fibres. The thickness was determined by making intensity line profiles across the lipid rich layer and measuring the full width half maximum (FWHM) of the profile (Fig. 1C,F). The thickness of the layer was found to be 0.93 ± 0.1 µm (N = 21) in MAS fibres and around 0.65 ± 0.1 µm (N = 15) in MiS fibres. Both values are well resolvable with CARS microscopy (resolution ≈ 300 nm). As no sample preparation or labeling is used in CARS microscopy the CARS signal is solely due to the lipid containing layer. Further support for the assumption of a thick lipid layer is given by deposited lipids found on the microscope cover glass where the fibres had made contact. Measuring the lipid layer thickness in hydrated samples showed no statistical difference. The size difference compared to previous work might be due to the fact that the investigated particular spider species produces thicker lipid layers. Alternatively, it could be speculated that the sample preparation reported in Ref.^6^ led to a modification of the original fibres, i.e., a removal of lipids. It should be noted that the apparent thickness of the layer could be influenced by structural irregularities in the fibre's outer layers.

Throughout the center of the fibre a weaker CARS signal was also observed (shown in Fig. 1A,C) that is thought to represent the CH2-bonds within the proteins. The autofluorescence, caused by two-photon excitation, was detected simultaneously with the CARS signal. Overlaying the two images (Supplementary Figs. S1 and S2) strongly suggests that it is the protein layers that emit autofluorescence in Fig. 1B,E).

CARS microscopy determined that MAS and MiS fibres consist of an inner autofluorescent protein core surrounded by a thick outer lipid layer. It was also found that the MAS fibres studied had a diameter of 13.8 ± 1.5 µm (N = 20, 5 different samples), while MiS fibres had a diameter of around 5.5 ± 0.5 µm (N = 23, 5 different samples), which is within the ranges previously reported^36^.

To image the outermost lipid containing layer of MAS and MiS fibres separately from the proteins, and to validate our CARS results, the fibres were labeled with both a lipophilic dye TopFluor PC and a more water-soluble fluorophore Rhodamine B. TopFluor PC is an amphiphilic molecule and has its fluorescent marker placed on the hydrophobic tail of the lipid molecule. This will mark the lipophilic layers. Since the protein layers of the fibres are thought to be more hydrophilic, the water-soluble fluorophore Rhodamine B was used for subsequent imaging.

Lipid dye labeling visualized the outermost lipid containing layers of both MAS and MiS fibres (Figs. 1G,J, green). The thickness of the lipid containing layer was found to be around 700 ± 300 nm for both MiS and MAS fibres. This is well within the resolution capability of the microscope (200–300 nm) and is also comparable to the lipid containing layer thickness found with CARS microscopy, considering the error bars. The images of the MAS fibres show that there is considerable variation in the thickness of the lipid rich layer, even along a single fibre. This could be a natural variation or due to the lipids being removed by contact with surfaces during collection and sample preparation.

As expected, Rhodamine B labels the inner protein layers (Fig. 1G,J, magenta). An area with low labeling between the two dyes was significantly larger in the MAS fibres (1.6 ± 0.3 µm) than in the MiS fibres (300 ± 300 nm). A possible explanation of this would follow the previous observation that the protein core of MAS fibres is covered by an outer protein layer^14^, so Rhodamine B could have lower affinity towards the proteins in this layer compared to the proteins within the core.

Another explanation would invoke super contraction during fibre wetting^18–20^. This was ruled out by a comparative experiment that used a different dye pair namely the hydrophobic dye Rhodamine B DHPE and the hydrophilic dye FITC. In this case, the hydrophilic dye, FITC, was again found to label the inner protein layers (Fig. 1H,K, magenta). In contrast to Rhodamine B, the hydrophilic dye FITC labeled all protein layers within the fibres without any area of low labeling. However, the fluorescence intensity towards the protein core decreases, showing that FITC has higher affinity towards the outer protein layers. During labeling the fibres were again submerged in water but no layer separation was observed for either MAS or MiS fibres, which rules out super contraction as the driving force for the labelling gap seen with Rhodamine B. We observed that Rhodamine B DHPE labeled the lipid layers successfully (Fig. 1H,K, green). The thickness of the lipid layers was found to be 600 ± 300 nm in MiS fibres and over 1 ± 0.3 µm in MAS fibres, which is in excellent agreement with the CARS measurements.

To investigate the lipid packing of the spider silk lipids, Laurdan GP measurements were performed. The GP value of MAS fibres was found to be 0.40 ± 0.02 (N = 5). This corresponds to a liquid-ordered or gel phase (Fig. 1I) which is in agreement with the assertion that the lipid layer of fibres consists mainly of highly hydrophobic lipids with a chain length between C28 and C34^7^. The GP value of MiS fibres was found to be 0.33 ± 0.05 (N = 5), corresponding to a more liquid-ordered phase (Fig. 1L). MiS fibres are used as temporary scaffolds during the construction of an orb web and therefore do not need to withstand external forces as much as MAS fibres. This could explain the lipids being in a liquid-ordered phase rather than a gel phase.

To resolve structures smaller than the present optical resolution limit of 200 nm such as protein fibrils, the fibres were imaged using Helium ion microscopy, combined with ion sputtering^30,32,33^.

**Figure 2:**
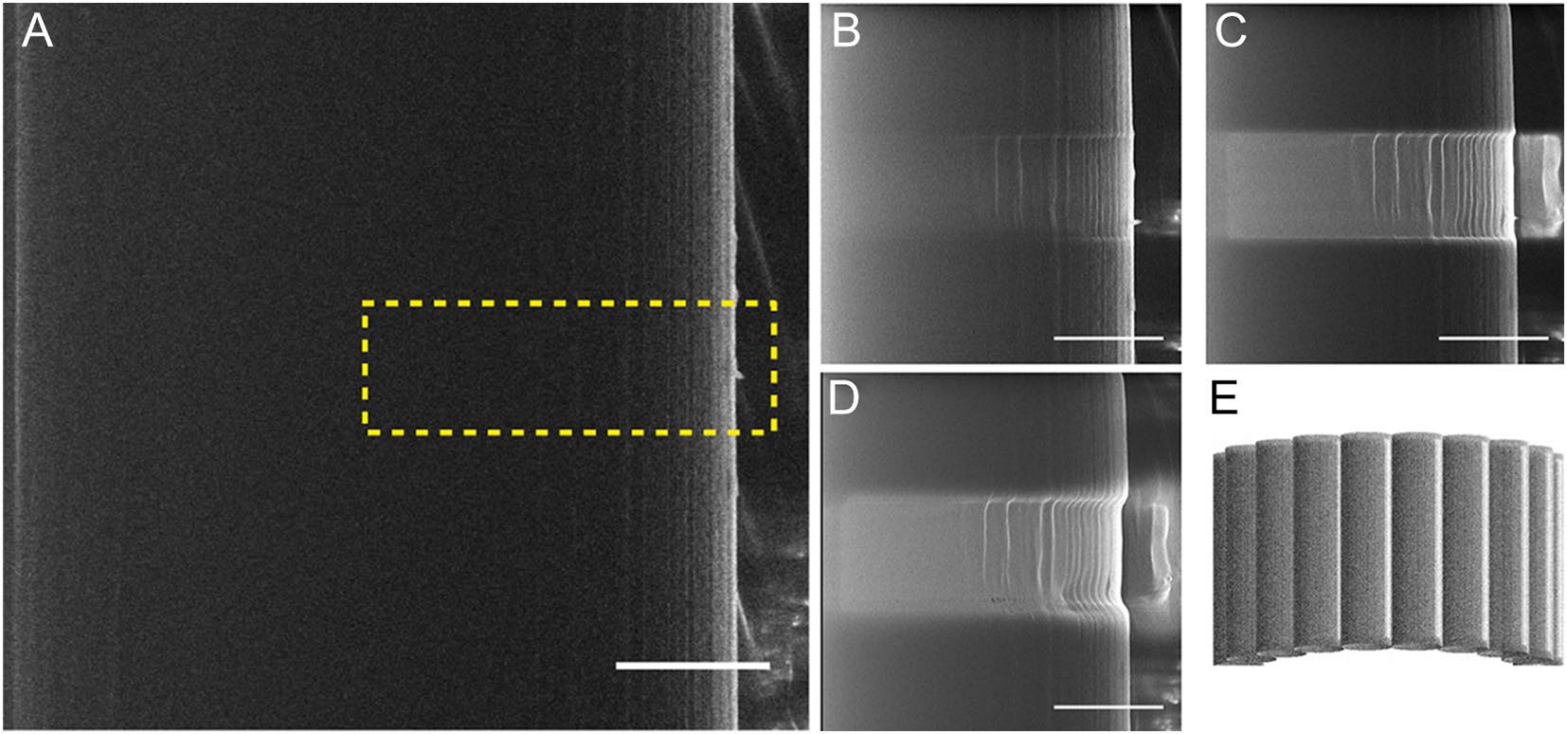
Scanning HIM images of a MAS fibre where the top layers are etched away perpendicularly to the fibre’s long axis using, He ion sputtering. (**A**) A spider silk fibre before etching in the marked area. (**B**–**D**) The top layers of the fibre from (**A**) are gradually being etched and fibrils within the fibre start to appear. VD = 8.5 mm, FoV = 6.5 µm and beam current = 0.284 pA. Scalebar is 2 µm. (**E**) Schematic representation of how fibrils appear in HIM.

**Figure 3:**
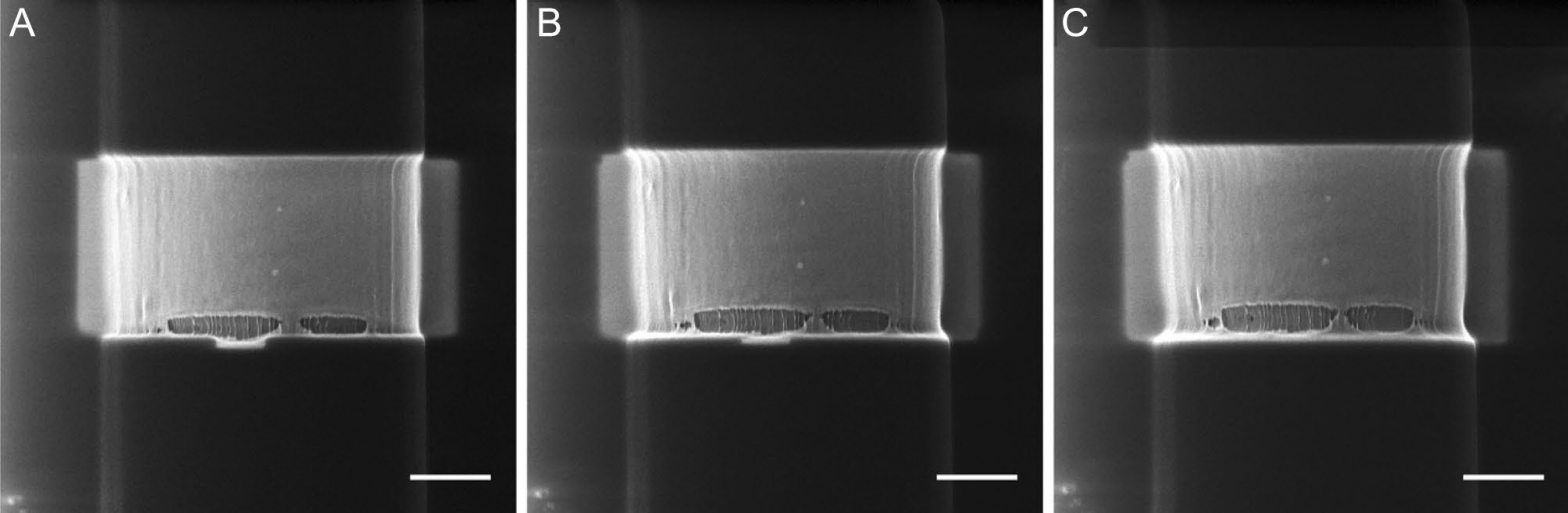
Scanning HIM images of a MiS fibre where the top layers are etched away consecutively by, He ion sputtering. The fibre shown have a diameter of the fibre of 4 µm. VD = 8.5 mm, FoV = 6.5 µm and beam current = 0.284 pA. Scalebar is 1 µm.

Figure 2A shows a MAS spider silk fibre with its edge on the right-hand side as the bright line. The diameter of the fibre was 9.5 µm. The surface of the fibre appears black due to surface charging. However, the edge of the fibre appears much brighter since more secondary electrons can escape from the edges. To image the fibrils within the fibre the outer most layers were removed via surface sputtering in the marked area in Fig. 3A. The sputtering was done using a He ion beam and the scanning direction of the beam was from left to right (across the fibre) in Fig. 2. The remaining images (Fig. 2B–D) show the same silk fibre but with the outer layers being sequentially removed throughout the images. Apparently, the fibrils run parallel to the long axis of the *Nephila* fibre, which coincides with a model proposed previously and confirmed recently using AFM^12,13^. Due to the edge effect the signal is thought to come from the more amorphous edges of and between the fibrils as depicted in Fig. 2E. Figure 2B–D show the brightness of the center of the sample increasing compared to Fig. 2A as charging decreases. This indicates that the outer lipid layers of the fibres are less conductive than the inner protein core.

We have also observed that the distance between the edges of the fibrils increases towards the center of the fibre. This is a geometrical effect, visualized by a simple schematic representation in Fig. 2E, which shows that the fibril diameter can be measured at the top of the fibre. The spacing between adjacent fibrils was measured for multiple fibres by determining the distance between the edge lines at the top of the fibre image, resulting in a width of 230 nm ± 22 (standard deviation, N = 11). This width is considerably smaller than the fibril diameter measured in Ref.^20^, which was on the order of 1 µm. However, the fibres in Ref.^20^ were super contracted, which could have also caused swelling in the fibrils.

**Figure 4:**
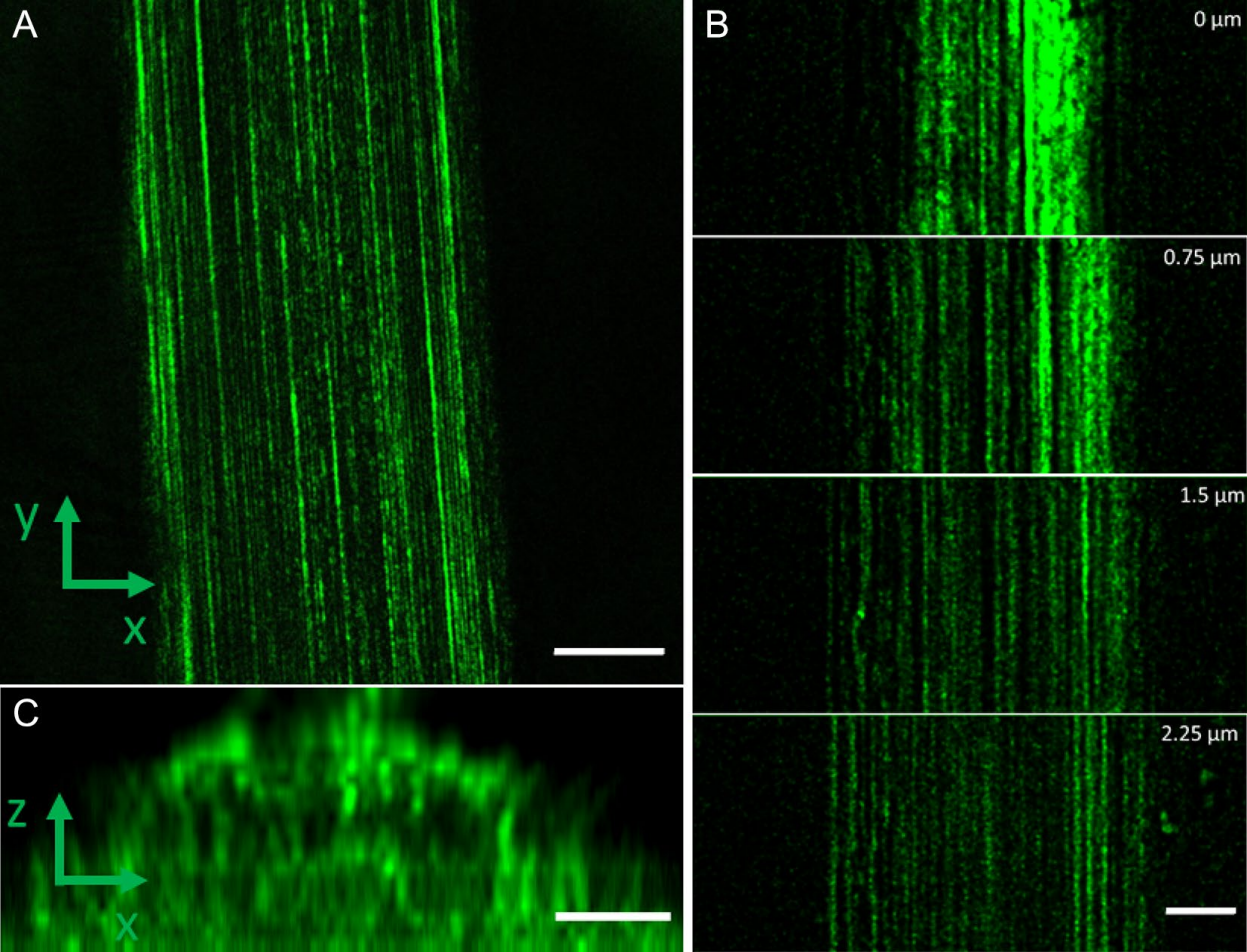
CRFD microscopy images of MAS spider silk fibres (**A**) Optical slice taken at the middle of a MAS fibre. Scalebar is 5 µm. (**B**) Optical sections of MAS fibre taken at different depths through the fibre. The depth is given by the number in the upper right corner of the images. Scalebar is 2 µm (**C**) Acquisition of images at different depths in z throughout the sample enabled reconstruction of a slice through the fibres in the xz plan, showing the fibre cross section. Scale bars 2 µm.

HIM was performed also on MiS fibres (Fig. 3). The surface of these fibres also appears black due to surface charging. The outermost layers are consecutively removed in order to image the fibrils. The etching area appears bright in Fig. 4A–C. As in MAS fibres, MiS fibrils appear as the surface layer is removed, and aligned parallel to the fibre’s long axis (Fig. 3A–C). However, at the bottom of the etched area a ripping of the layer is visible. In this area more fibrils can be observed with much brighter edges. The HIM images consequently show two distinct inner layers in MiS fibres. It may therefore be that the second layer removed was a skin layer as proposed by Sponner et al.^6^. The fibril spacing was 99 nm ± 27 (standard deviation, N = 12). These images show that the fibrils of MiS fibres have a smaller diameter than those of MAS fibres, suggesting that the overall size of the fibre affects the size of the fibrils. This assumption is supported by previous AFM images of 2 µm wide spider silk which showed surface fibrils of the order of 40 nm^12^. Additionally, studies in different spider species also demonstrate considerable variation^37^.

Whereas HIM results in unprecedented detail in scanning mode, it is a technique that requires the sample to be kept in a vacuum during imaging. It is also a very surface-sensitive technique which prevents imaging of the structures within the fibres. A different technique must be used to image the protein core's design in intact, pristine fibres. As the protein core within the fibres contains crystalline regions^38–41^, a strong optical scattering signal is expected that could be imaged by, e.g., using CRFD microscopy (Fig. 4).

**Figure 5:**
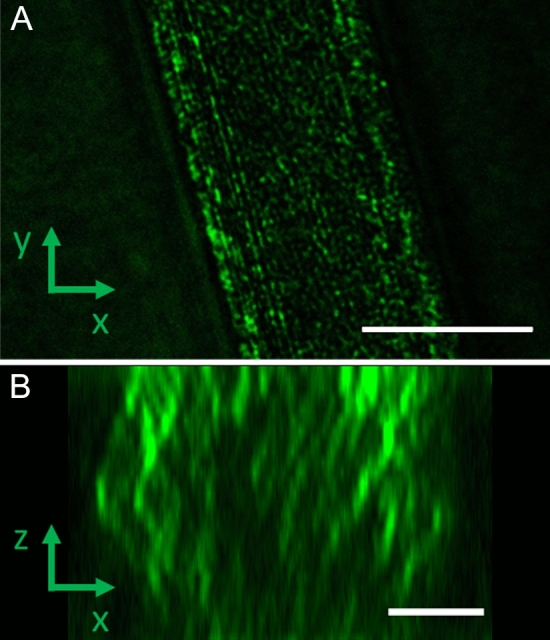
CFRD microscopy images of MiS spider silk fibre. (**A**) Optical slice taken at the middle of a MiS fibre. Scalebar is 5 µm. (**B**) Acquisition of images at different depths in z throughout the sample enabled the reconstruction of a slice through the fibres in the xz plan, showing the fibre cross section. Scale bars 2 µm.

CRFD microscopy enables visualization of fibrils in a pristine, untreated fibres. The fluorescence depletion laser enhances contrast by reducing autofluorescence from the sample. Upon examination of optical sections in both MAS (Fig. 4) and MiS fibres (Fig. 5), fibril-like structures were observed throughout the protein core. Furthermore, the technique allows imaging of optical slices through the fibre, making it possible to reconstruct cross-sections (Figs. 4C, 5B) and visualize the fibrils throughout the fibre. The ends of the fibrils appear elongated in the Z direction due to the lower resolution in this direction compared to the XY direction. In the cross sections the fibrils can be seen to be distributed throughout the fibre.

These fibrils were found also to be arranged parallel to the fibre’s long axis. The fibrils in the MiS fibres are seen to be more punctuated and have a smaller diameter (116 nm ± 12 (SD, N = 14)) than the MAS fibre 145 nm ± 18 (SD, N = 14). Note that the resolution limit of CRFD microscopy is on the order of 110 nm, meaning that the MAS structures are most likely resolved while the structures in the MiS images could be smaller than the resolution of the technique.

**Figure 6:**
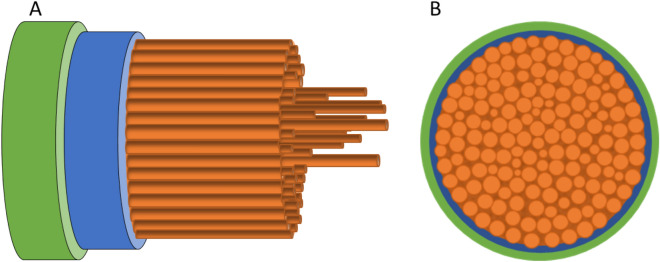
Schematic representation (not to scale) of the proposed structure of a spider silk fibre as found in the present work. (**A**) Fibre side view, (**B**) cross section through fibre. An outer, non-conductive lipid rich layer (green) of between 0.6 to 1 µm thickness, two conductive, inner autofluorescent protein layers: one which FITC shows higher affinity towards (blue), and another Rhodamine B shows a higher affinity towards (orange). The inner protein core consists of crystalline fibrils, aligned parallel to the long axis of the fibre, surrounded by more amorphous protein regions.

Table 1 shows measurements for the fibral spacing and diameter as measured by HIM and CRFD. For MAS fibrils both HIM and CRFD give similar fibril spacing on the order of 240 nm with a fibril diameter of 145 ± 18 nm. The fact that the HIM measurements presented do not show voids suggests a structure with crystalline fibrils arranged parallel to the fibre long axis surrounded by areas of less scattering amorphous proteins. This implies that the dark space between the fibres in CRDF are more amorphous areas with lower scattering as suggested by Shao et al.^42^ This interpretation is supported by previous polarized two-photon excitation microscopy results, showing that the fibres consist of a distribution of crystalline and amorphous proteins^39^. Figure 6 provides a simple sketch of the structural model of silk fibres described here.

**Table 1:** The measurements of fibral spacing and diameter.

	Fibril spacing HIM (nm)	Fibril spacing CRFD (nm)	Fibril dia. CRFD (nm)
MAS	230 ± 22 (N = 11)	247 ± 49 (N = 33)	145 ± 18 (N = 14)
MiS	99 ± 27 (N = 17)	206 ± 34 (N = 8)	116 ± 12 (N = 14)

In the case of MiS fibres, the spacing of the fibrils measured by HIM and CRFD is quite different (see Table 1). This is most likely due to the small size of the fibrils and spacing which are below the resolution limit of the CRFD. Therefore, the spacings which can be resolved in CRFD are typically all larger than 99 nm as measured by HIM.

## Conclusions

This work examined the micro- and nanostructures of spider silk fibres from the orb-weaving spider *Nephila madagascariensis* using several novel microscopy techniques.

The results of CARS, Laurdan, and confocal microscopy revealed that both MAS and MiS fibres exhibited a lipid-rich outer region with a thickness of over 500 nm. This is thicker than the values reported by Sponner et al.^6^. We note that the silks used here were pristine and untreated while the lipid layer identified by Sponner appeared to be detaching from the fibre due to the sample preparation and labeling procedure used. However, it is important to note that the present experiments only show a lipid rich hydrophobic region, not a pure lipid layer. This means that this region could well consist of outer lipid layers followed by a mixed lipid glycol protein layer as suggested by Ref.^6^. Further support for this interpretation comes from the HIM images which show emerging fibres after just 100–200 nm of surface sputtering.

Hydrophilic dyes such as Rhodamine B and FITC label the inner protein layers of the fibres. Rhodamine B was found to have a higher affinity towards the inner core proteins as almost no labeling was observed towards the outer layers but a high amount of labeling within the center of the fibres. However, FITC had increased labeling towards the edges showing that this dye has a higher affinity towards the proteins in the outer layers. The different affinities of the dyes suggest that the fibres have an outer and inner protein layer with different physical chemical properties (Fig. 6).

The protein core structure in MAS and MiS fibres was examined using scanning HIM and CRFD microscopy techniques. Both HIM and CRFD microscopy revealed that the protein cores of MAS and MiS fibres consist of fibrils aligned parallel to the long axis of the fibre. Fibril diameters were found to vary between 145 nm ± 18 and 116 nm ± 12 depending on the fibre and fibre type, suggesting that the fibres exhibit considerable natural diversity. This is supported by recent results using AFM^17^ and SEM^43^. Furthermore, studies in different spider species also show considerable variations^37^.

From the HIM images two distinct inner layers could also be observed in MiS fibres: one layer appearing after removal of the lipid layer and another appearing after removal of the first layer. This indicates a multilayered structure of the silk fibre. While HIM could only image the outermost fibrils, CRFD microscopy was able to visualize parallel crystalline protein fibrils oriented along the fibre axes throughout the whole fibre surrounded by less scattering and more amorphous protein structures.

The nanoscopic fibril structure and organization found in this work demonstrates the importance of studying samples at multiple size scales, not just the molecular structure and macroscopic properties, but also the nanoscopic structures linking the two. The relevance of the nanoscopic structures for spider silk’s macroscopic properties still needs further studies. However, we assert that these hierarchical structures will be highly relevant in any attempt to consolidate structure function relationships. Taking these kinds of studies further will also be crucial when trying to create and analyze artificial silk inspired polymer filaments.

## Methods

Phosphate Buffered Saline (PBS) was prepared from tablets (Sigma-Aldrich, Denmark). One tablet dissolved in 200 mL of MilliQ water yielded 0.01 M phosphate buffer with 0.0027 M potassium chloride and 0.137 M sodium chloride, pH 7.4, at 25 °C.

All other chemicals used were from Sigma-Aldrich (Denmark). The fluorescent dyes used in confocal microscopy were: 1-palmitoyl-2-(dipyrrometheneborondifiuoride)undecanoyl-sn- glycero-3 phosphoethanolamine, TopFluor PC (Avanti Polar Lipids, USA), Rhodamine B (Sigma-Aldrich, Denmark), Fluorescein isothiocyanate, FITC (Thermo Fischer Scientific, USA) and Rhodamine B DHPE (Sigma-Aldrich, Denmark). The embedding medium used in confocal microscopy and GSD-Raman was Pro-Long Diamond Slowfade reagent (Life Technologies, Denmark).

### Silk extraction

Spider silk fibres for all experiments were drawn from 2 different female Nephila madagascariensis (BugzUK, United Kingdom) spiders by forceful silk extraction. Twice a week, the spiders were fed mealworms and crickets ad libitum.

Firstly, the spider was placed on a Styrofoam surface and covered by tulle. The tulle was then fixed to the Styrofoam surface with pins resulting in the tulle exerting stress on the spider immobilizing it. A hole was made in the tulle to allow access to the spinnerets. After having immobilized the spider, MAS fibres were pulled out of the Major Ampullate gland by making contact with the spinneret with a tweezer and pulling gently. The silk fibre was then fastened to a reeling machine using double-sided tape. MiS fibres were reeled out with MAS fibres but separated with tweezers in order to collect only MiS fibres and then fastened to a reeling machine. The spider silk was reeled out at a constant speed of 7.7 mm s^−l^.

### CARS microscopy

CARS images of MAS and MiS fibres were imaged using a Leica SP8 (Mannheim, Germany). The pump laser used was a picosecond pulsed laser at 816 nm (symmetric CH_2_-stretch at 2850 cm^−l^), and the Stokes laser was also a picosecond laser at 1064 nm. Autofluorescence was excited by the pump laser (816 nm) and imaged simultaneously.

Two non-descanned PMT detectors were used, one for CARS and one for autofluorescence measurements, both in the epi-direction. A bandpass filter (661 nm ± 5.5) was placed in front of the CARS detector, and a bandpass filter (475 nm ± 100) was placed in front of the autofluorescence detector. Images were collected during scans with the galvo scanners. A 40× IRAPO water objective lens with a numerical aperture of 1.1 was used for all measurements. The samples were mounted on cover slides and the coverslips were mounted using nail polish.

The thickness of the lipid-rich layer in the fibres was measured by analyzing the intensity profile across the layer and determining the full-width at half-maximum (FWHM) of the peak. These measurements were carried out using FIJI software^44^.

### Laurdan GP measurement

For Laurdan GP measurements MAS and MiS fibres were labeled with an 8 µM Laurdan in milliQ water solution and mounted in milliQ water on cover slides. The coverslips were then mounted using nail polish. The microscopy and imaging set-up was done as described in Ref.^45^. After acquiring the intensity distributions, the GP images were computed with SimFCS software (Laboratory for Fluorescence Dynamics, Irvine, CA). The GP images were calibrated with a correction factor g obtained from a Laurdan GP standard (4 µM Laurdan in DMSO). The Laurdan GP value of the reference solution was measured in an ISS Chronos spectrofluorometer (ISS, Champaign, USA) and it was found to be 0.019.

### Confocal microscopy

Confocal images of MAS and MiS fibres were imaged using a Leica SP8 (Mannheim, Germany). The excitation laser used was a pulsed white light laser. The system was equipped with a hybrid detector and gated detection. Images were collected during scans with galvo scanners. A 100× oil objective (NA = 1.40) lens was used in all measurements.

Silk samples for confocal imaging were labeled using the following dyes with a concentration of 4 µM: Rhodamine B (PBS buffer), TopFluor PC (PBS buffer), Rhodamine B DHPE (MeOH) and FITC in DMSO. The structure and absorption and emission spectrum for each dye can be seen in Supplementary Tables S1–S4. The samples were labeled in the following dye pairs: Rhodamine B + TopFluor PC; Rhodamine B DHPE + FITC. The samples were mounted onto cover slides and then the coverslips were mounted using Prolong Diamond SlowFade as embedding medium.

### Scanning HIM

MAS and MiS fibres were imaged using a Zeiss ORION NanoFab Helium Ion Microscope with SE detection and without metallic coating. Charge compensation was ensured through a low-energy electron beam (flood gun, 600 eV) directed at the sample. Surface sputtering was performed at 25 keV beam energy, with a probe current ranging from 2 to 10 pA. During the cutting the flood gun was continuously on. The silk was cut into a length to fit on top of aluminum specimen mounts (Plano GmbH, Germany) and mounted using carbon tape (Ted Pella, Inc., USA).

### Confocal reflection fluorescence depletion (CRFD) microscopy

The basis for CRFD microscopy is confocal reflection microscopy combined with a reduced pinhole size which has been shown to improve the resolution of the confocal microscope up to 1.38 times the diffraction limited resolution^23,25–27^. Furthermore, while the sample is scanned with the laser light that generates the reflected signal, it is simultaneously illuminated by another overlapped toroidal shaped laser beam, which depletes the inherent autofluorescence in the sample and thus creates better contrast^24^.

CRFD images of MAS and MiS fibres were imaged using a Leica SP8 (Mannheim, Germany). The excitation laser used was an Argon laser at 458 nm. The depletion laser was a CW laser at 592 nm. The system was equipped with a hybrid detector and used non-gated detection around 496 nm. Images were collected during scans with galvo scanners. A 100× oil objective (NA = 1.40) lens was used in all the measurements. The pinhole size was set at 0.6 AU.

The samples were mounted onto cover slides and the coverslips were mounted using Prolong Diamond slowfade. The thickness of the fibres was found by measuring the diameter of the imaged protein core and adding 1 µm as the thickness of the lipid layer.

## Supplementary Information

Supplementary Information.

## Data Availability

The datasets used and/or analyzed during the current study are available from the corresponding author on reasonable request.
