# Supplementary material for: Nanoscale imaging of major and minor ampullate silk from the orb-web spider Nephila Madagascariensis
Source: Sci Rep. 2023 Apr 24;13:6695. doi: 10.1038/s41598-023-33839-z (PMC10125981; doi:10.1038/s41598-023-33839-z)
Supplement: Supplementary file 1 — Supplementary Information. [file 41598_2023_33839_MOESM1_ESM.docx]

**Supplementary Material**

**Nanoscale imaging of major and minor ampullate silk from the orb-web spider Nephila Madagascariensis**

Irina Iachina^1,2^, Jacek Fiutowski^1^, Horst-Günter Rubahn^2^, Fritz Vollrath^3^, and Jonathan R. Brewer^1*^

^1^ Department of Biochemistry and Molecular Biology, University of Southern Denmark, Odense, Denmark

^2^ Mads Clausen Institute, SDU NanoSYD, University of Southern Denmark, Sønderborg, Denmark

^3^ Department of Biology, University of Oxford, South Parks Rd., UK

* Corresponding author: E-mail: [brew](mailto:brewer@bmb.sdu.dk)[er@bmb.sdu.dk](mailto:er@bmb.sdu.dk)


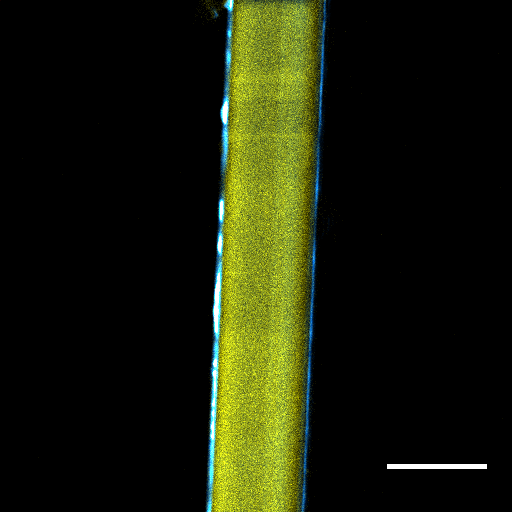


Figure S1: ﻿Overlay of a CARS image of CH_2_-stretch at 2850 cm^-1^ (blue) and autofluorescence (yellow) in a MAS fiber from the orb-weaver spider *Nephila madagascariensis*. Scalebar is 10 μm.


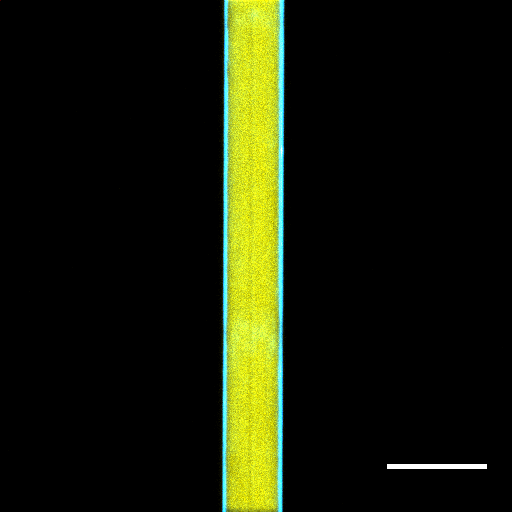


Figure S2: ﻿Overlay of a CARS image of CH_2_-stretch at 2850 cm^-1^ (blue) and autofluorescence (yellow) in a MiS fiber from the orb-weaver spider *Nephila madagascariensis*. Scalebar is 10 μm.

Table S1. Structure and absorption and fluorescence spectrum of the fluorescent dye TopFluor PC.

| **Dye** | TopFluor PC |
| --- | --- |
| **Structure** | 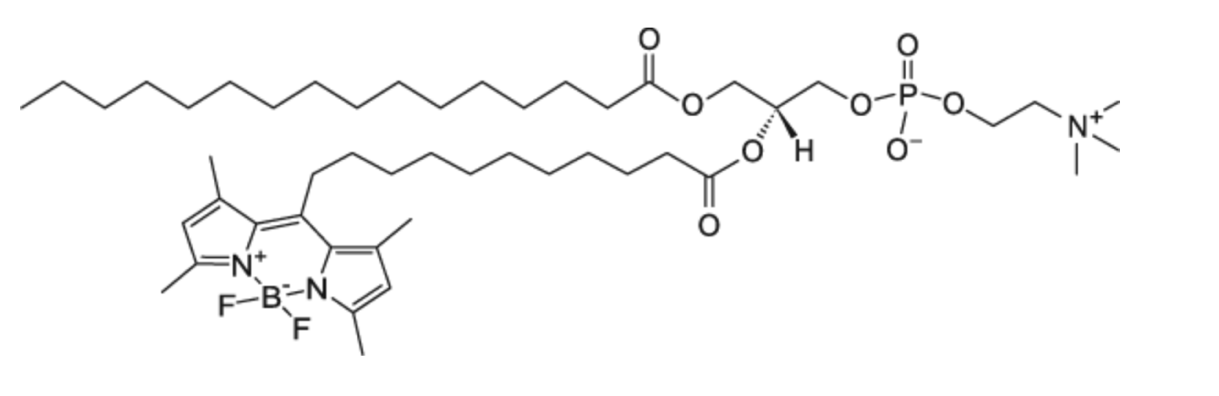 |
| **Spectrum** | 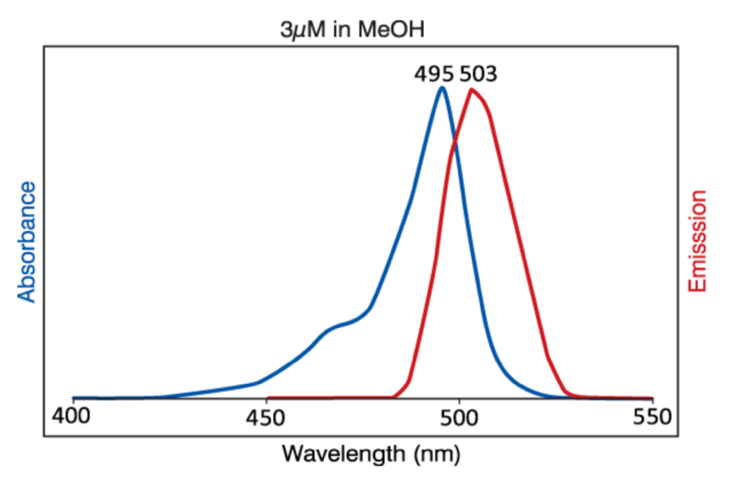 |

Table S2. Structure and absorption and fluorescence spectrum of the fluorescent dye Rhodamine B DHPE

| **Dye** | Rhodamine B DHPE |
| --- | --- |
| **Structure** | 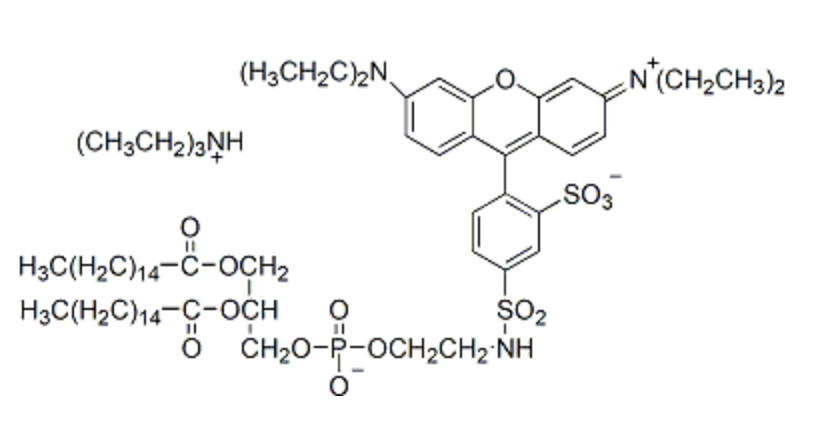 |
| **λ_ex_/λ_em_**  **Spectrum** | 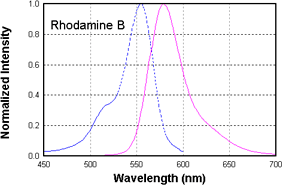 |

Table S3. Structure and absorption and fluorescence spectrum of the fluorescent dye Rhodamine B.

| **Dye** | Rhodamine B |
| --- | --- |
| **Structure** | 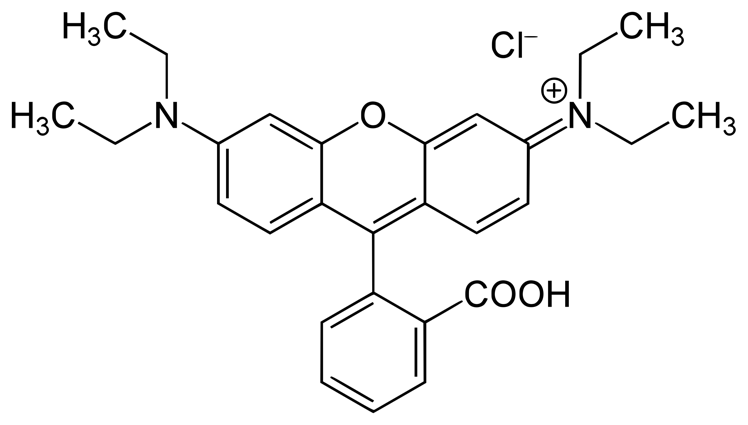 |
| **Spectrum** | 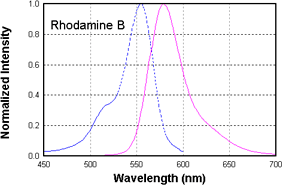 |

Table S4. Structure and absorption and fluorescence spectrum of the fluorescent dye FITC.

| **Dye** | Fluorescein isothiocyanate, FITC |
| --- | --- |
| **Structure** | 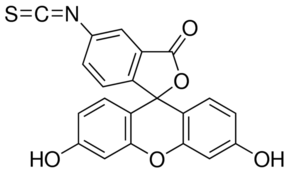 |
| **Spectrum** | 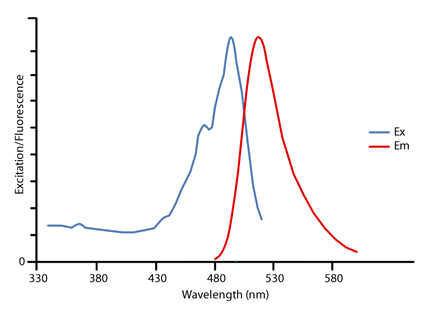 |
